# Identification and characterization of the *GhHsp20* gene family in *Gossypium hirsutum*

**DOI:** 10.1038/srep32517

**Published:** 2016-09-01

**Authors:** Wei Ma, Ting Zhao, Jie Li, Bingliang Liu, Lei Fang, Yan Hu, Tianzhen Zhang

**Affiliations:** 1 National Key Laboratory of Crop Genetics and Germplasm Enhancement, Cotton Hybrid R & D Engineering Center (the Ministry of Education), Nanjing Agricultural University, Nanjing, 210095, China

## Abstract

In higher plants, Heat Shock Protein 20 (*Hsp20*) plays crucial roles in growth, development and responses to abiotic stresses. In this study, 94 *GhHsp20* genes were identified in *G. hirsutum*, and these genes were phylogenetically clustered into 14 subfamilies. Out of these, 73 paralogous gene pairs remained in conserved positions on segmental duplicated blocks and only 14 genes clustered into seven tandem duplication event regions. Transcriptome analysis showed that 82 *GhHsp20* genes were expressed in at least one tested tissues, indicating that the *GhHsp20* genes were involved in physiological and developmental processes of cotton. Further, expression profiles under abiotic stress exhibited that two-thirds of the *GhHsp20* genes were responsive to heat stress, while 15 genes were induced by multiple stresses. In addition, qRT-PCR confirmed that 16 *GhHsp20* genes were hot-induced, and eight genes were up-regulated under multiple abiotic stresses and stress-related phytohormone treatments. Taken together, our results presented here would be helpful in laying the foundation for understanding the complex mechanisms of *GhHsp20* mediated developmental processes and abiotic stress signaling transduction pathways in cotton.

Arguably, increased yield could be best achieved by selecting genes for increased yield under optimal production conditions. Plants with higher yields in high inputs environment are more likely to have higher yields under stressed conditions^1^.

Abiotic stresses, such as high temperature, low temperature and drought, influence plant growth and development. The stress signals produced can stimulate plants to synthesize a series of responsive proteins to protect their cell metabolism. Heat Shock Proteins (Hsps) are important type of stress-induced proteins that are produced in plants in response to external stresses. Hsps exist in a wide variety of organisms and biospheres. When tissues or cells deal encounter various stresses, heat shock transcription factor (HSF) binds to the heat shock element (HSE) in the upstream region of Hsp to increase Hsp expression^2^. Increased Hsp expression strengthen the ability of plants to resist various stress factors, as it acts as a molecular chaperone that has a significant role in stress physiology. According to their molecular weights and amino acid sequence homology, Hsps can be classified as high molecular mass proteins, including Hsp100, Hsp90, Hsp70/DnaK and Hsp60/GroE, and low molecular mass proteins, including Hsp20, a type of small heat shock protein (sHsp)^2^.

The Hsp20 proteins are the most abundant heat shock proteins found in plants, and experiments have shown that this group plays an important role in the heat tolerance of plants, providing plants with at least a temporary protection mechanism. In plants, Hsp20 proteins are encoded by nuclear multigene families and are localized in different cellular compartments. Hsps have been classified into 14 subfamilies; nine of these are localized to cytoplasm or nucleus (CI–CXI) and five are localized in organelles. The organelle subfamilies include one localized to peroxisomes (PX), another to chloroplasts (CP), one to the endoplasmic reticulum (ER), and two to the mitochondria (MTI and MTII). Ten of these subfamilies exist in both monocots and eudicots (CI, CII, CIII, CIV, CV, PX, CP, ER, MTI, and MTII), and one is found only in eudicots (CVI)^2^,^3^,^4^. In total, 19 genes encoding Hsp20 have been identified in *Arabidopsis*, and these are grouped into 12 subfamilies based on their subcellular localization and homology, while 23 Hsps have been identified in *Oryza sativa*^2^,^3^,^4^,^5^,^6^. With other two subfamilies described in other species, a total of 16 subfamilies have been identified in plants^3^,^4^,^5^,^6^,^7^.

The alignment of Hsp20 gene sequences cloned from soybean, pea, *Arabidopsis*, carrot, wheat, corn, tomato and other plants suggests that the Hsp20 proteins are relatively conserved in plants. The main characteristic of Hsp20 proteins is a highly conserved 80–100 amino acid sequence called the alpha crystalline domain (ACD), located in the C-terminal region. This domain is divided into two conserved regions by a hydrophobic region of variable length, N-terminal consensus I (27 amino acids) and C-terminal consensus II (29 amino acids)^8^,^9^,^10^. The Hsp20 proteins are ATP-independent molecular chaperones that usually spontaneously form large oligomeric complexes ranging in size from 9 to 50 subunits (200–800 kDa) and act by preventing protein denaturation in both eukaryotic and prokaryotic cells^11^,^12^.

Recently, researchers have demonstrated that Hsp20 proteins function as molecular chaperones and play an important role in plant immunity by inhibiting apoptosis^13^, promoting cytoskeleton formation, and protecting the mitochondrial and PS II electron transport chain. For example, SHsp transgenic tomato showed less electrolyte leakage, chlorophyll damage and accumulation of anthocyanin than that in wild type under low temperature stress^14^. *AtHSP17.6A* overexpressed in *Arabidopsis thaliana* was found to increase salt and drought tolerance^15^. *RcHSP17.8* from *Rosa chinensis* was overexpressed in Escherichia coli and yeast, and these cells showed improved viability under thermal, salt and oxidative stress^16^.

Cotton is an important fiber crop that provides lint for textile industry and oil for edible purposes, but its growth, yield and fiber quality are greatly affected by various abiotic stresses, such as drought, salinity and high temperature. Therefore, improving stress tolerance in cotton cultivars is a priority for most cotton breeding programs. Previous research has shown that Hsps are ideal targets for improving tolerance to a wide range of stresses. However, the Heat Shock Protein 20 (*GhHsp20*) family in cotton is largely unknown, and no *Hsp20* genes responsive to biotic stress have been identified in cotton. Thus it is imperative to study the *GhHsp20* family in cotton. Herein, genome-wide and comprehensive expression analyses of *GhHsp20* were performed. Our results will provide a foundation for understanding the functional structures and genomic organization of the *GhHsp20* gene family in cotton, and are undoubtedly useful in the detailed characterization of the function of these genes.

## Results

### Identification and sequence conservation of *GhHsp20* genes in *G. hirsutum*

A total of 111 genes were identified in the *G. hirsutum* genome as candidate members of the *GhHsp20* family. To further verify the reliability of these candidate sequences, the amino acid sequences of all 111 proteins were searched for the presence of the Hsp20 domain using Pfam and SMART software. InterPro analysis showed that the ACD (PF00011) was absent in 14 sequences, and 97 proteins possessed the principal ACD. A total of three of the 97 sequences contained two domains, and these were excluded from the subsequent analysis. In total, 94 typical *GhHsp20* genes were therefore identified from the original data. The encoded proteins varied from 113 to 268 amino acids in length. These 94 *GhHsp20* genes were subjected to further analysis; detail on the other parameters of the nucleic acid and protein sequences are provided in Supplementary Dataset 1.

**Figure 1:**
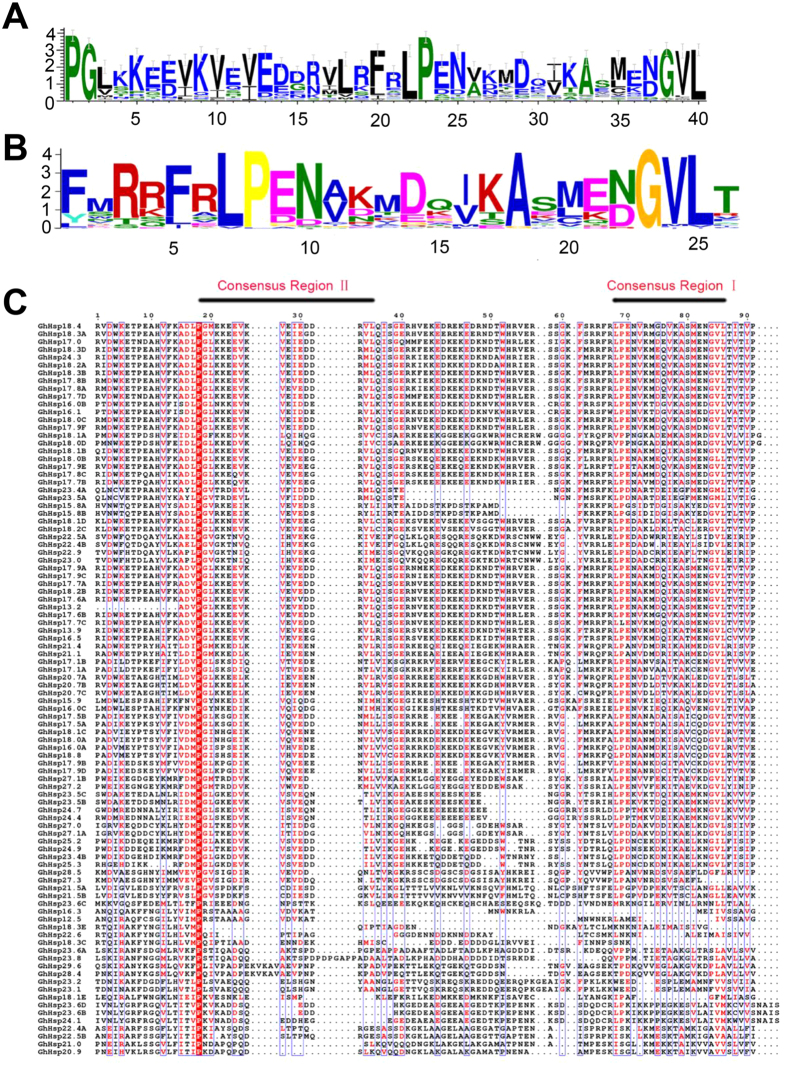
The ACD is highly conserved across all *GhHsp20* proteins. (**A**) Sequence logo of the *GhHsp20* domain in 94 cotton *GhHsp20* genes generated by the application WebLogo (http://weblogo.threeplusone.com). The heights of symbols within each stack indicate the relative frequency of each amino acid at that position. The logo was obtained through the multiple alignment of the *GhHsp20* domain in 94 cotton *GhHsp20* protein sequences. The sequence logos of the *GhHsp20* proteins were based on full-length alignments of all *GhHsp20* proteins. (**B**) HMM logo from Pfam representing the Hsp20 domain (PF00011). The overall height of each stack indicates the conservation of the sequence at that position, whereas the height of letters within each stack represents the relative frequency of the corresponding amino acid. (**C**) Multiple sequence alignment of the *GhHsp20* proteins. Gene identity numbers are provided on the left. Color shading indicates the types of amino acid residues that were conserved. The defined regions are underlined.

To explore the *GhHsp20* domain, sequence logo and alignment information were produced to examine how well-conserved the domains were in the *GhHsp20* proteins within each residue position. The sequence logo of the 94 *GhHsp20* genes shown in Fig. 1A was generated using the WebLogo application (http://weblogo.threeplusone.com)^17^. The multiple sequence alignment analysis and sequence logo revealed that all of the *GhHsp20* proteins shared regions of conserved polypeptide sequences, including consensus region I and consensus region II, which are involved in the function of molecular chaperones (Fig. 1C).

### Phylogenetic analysis of *GhHsp20* gene family

**Figure 2:**
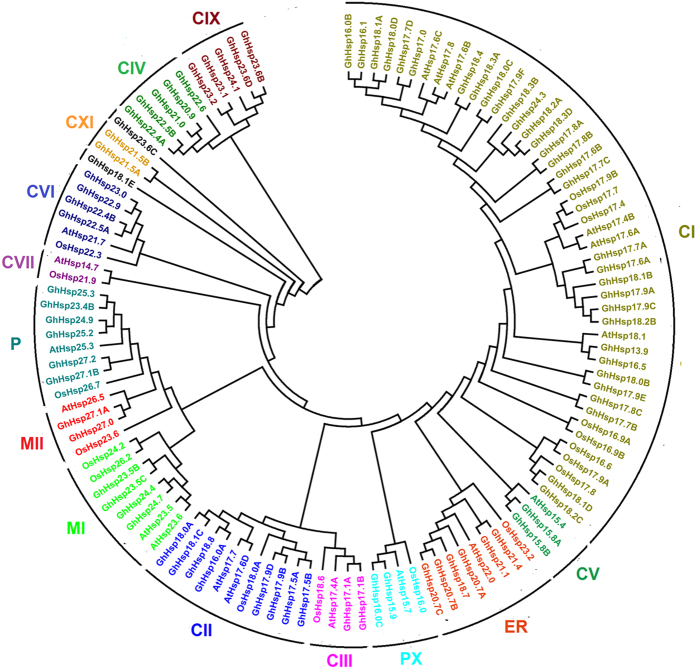
Phylogenetic tree of Hsp20 proteins from *G. hirsutum*, *Arabidopsis* and *Oryza sativa*. The deduced full length amino acid sequences were aligned by ClustalX 2.0 and the phylogenetic tree was constructed using MEGA 5.0 by the Neighbour-Joining (NJ) method with 1,000 bootstrap replicates. Each Hsp20 subfamily is indicated by a specific color.

*In order to analyze the evolutionar*y relationships of *Hsp20* genes and to help in their classification, the full length of *Oryza sativa Hsp20 (OsHsp20*), *Arabidopsis thaliana Hsp20 (AtHsp20*) and *G. hirsutum Hsp20 (GhHsp20*) were used to generate an unrooted phylogenetic tree. All the identified *GhHsp20* genes were classified into 14 subfamilies (Fig. 2). Based on the phylogenetic tree and in silico subcellular localization analysis, we identified *GhHsp20* members related to the previously defined CI, CII, CIII, CIV, CV, CVI, CVII, CIX, CXI, MI, MII, ER, P and PX subfamilies. Thus, the *GhHsp20* genes were distributed between a total of 13 subfamilies as follows: the nucleocytoplasmic subfamilies (C) contained eight subfamilies and constituted the largest clade, containing 60 members and accounting for 73.2% of the *GhHsp20* genes; the M subfamily contained two subfamilies, comprising a total of six *Hsp20* genes; six *Hsp20* genes belonged to the ER subfamily; six belonged to the P subfamily, and two belonged to the PX subfamily. Finally, two orphan genes (*GhHsp23.6C* and *GhHsp18.1E*) did not belong to any subfamily, possibly because of their apparently incomplete structures. As shown in the phylogenetic tree (Supplementary Figs 6 and 7), most of the ortholog genes between two diploids and allotetradiploid were clustered into a same clade.

Although evolutionary relationships could not be elucidated for all proteins, the analysis showed some interesting results. Noticeably, among the subgroups, three subfamilies (CVI, CIX and CXI) contained *Hsp20* genes from cotton only, and subfamily CVII only contained *Arabidopsis thaliana* and *Oryza sativa Hsp20* genes. Compared to the MI and MII clades, which contained similar numbers of proteins from each species, the CII clade contained considerably different numbers of proteins from each of the three species. This suggested that expansion of these subfamilies has occurred since the divergence between eudicots and monocots.

### Gene structure and conserved motif distribution analysis

**Figure 3:**
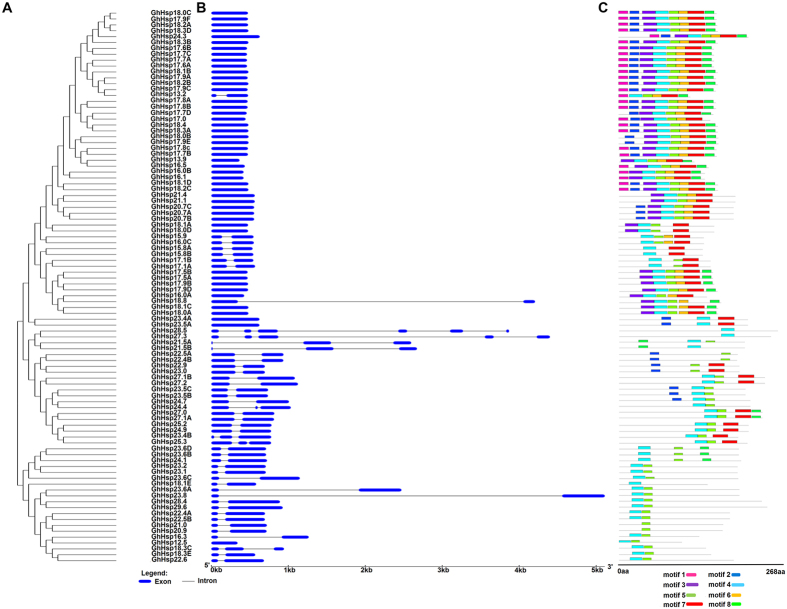
Phylogenetic relationships, gene structure and motif compositions of *GhHsp20* genes. (**A**) Multiple alignments of 94 full length *GhHsp20* proteins were conducted by Clustal X 2.0 and the phylogenetic tree was constructed using MEGA 5.0 by the Neighbor-Joining (NJ) method with 1,000 bootstrap replicates. The percentage bootstrap scores higher than 50% are indicated on the nodes. (**B**) Exon/intron organization of *GhHsp20* genes. Blue line represents exon and black line represents intron. The sizes of exons and introns can be estimated using the scale at bottom. (**C**) Schematic representation of the conserved motifs in *GhHsp20* proteins elucidated by SMART online. Each colored box represents a motif in the protein with motif name indicated in box at bottom. The length of the protein and motif can be estimated using the scale at bottom.

In plants, most genes are interrupted genes, with one or more exons and several introns. The arrangement of introns and exons can be used to analyze the evolutionary relationships among different gene family members. The genomic sequence of the longest *GhHsp20* gene (*GhHsp29.6*) was about 807 bp, while the shortest (*GhHsp12.5*) was only 339 bp. To gain a further insight into the possible structural evolution of *GhHsp20*, a separate unrooted phylogenetic tree was constructed using the protein sequences of all the *GhHsp20* genes (Fig. 3A). The exon/intron organization was then compared in the coding sequences of the *GhHsp20* genes (Fig. 3B). Figure 3B provided a detailed illustration of the relative lengths of introns and exons. A highly diverse distribution of exon regions (from one to six in numbers) was found among the *GhHsp20* genes. However, it was worth noting that closely related genes were generally more structurally similar, differing only in intron and exon lengths. In general, the 94 *GhHsp20* genes can be divided into three categories according to their intron numbers. Half of all gene family members (47 genes) had no introns. This value was far higher than the distribution ratio of genes with no introns in the genome as a whole. In addition, 39 genes had only one intron, and there were just eight genes with multiple introns. Our results indicated a strong correlation between the phylogeny and exon/intron structure of these genes, and the regularity of the gene structure may be associated with evolutionary trends, and may reflect the conservation in the gene families.

In total, eight motifs, named motifs 1 to 8 were identified. As shown in Fig. 3C, *GhHsp20* proteins in the same group contained similar motifs. Most members of the *GhHsp20* family shared two motifs, motif 4 and motif 5, which were linked in order. A few members, such as GhHsp22.5A, GhHsp22.4B, *GhHsp20*.9, and GhHsp21.0, showed quite different protein structures compared with the other members. Interestingly, motif 1 was selectively distributed among a specific subgroup (CI) in the phylogenetic tree. The unique motifs in different subfamilies may relate to the conservation and specific functions of the *GhHsp20* gene family. The clustered *GhHsp20* pairs, i.e. *GhHsp18.0C* and *GhHsp17.9F*, showed similar motif distributions. The motifs and their arrangement in the *GhHsp20* proteins were similar among proteins within the same subgroup, demonstrating that the protein architectures were remarkably conserved within a specific subfamily.

### Chromosomal location and gene duplication of *GhHsp20* in *G. hirsutum*

**Figure 4:**
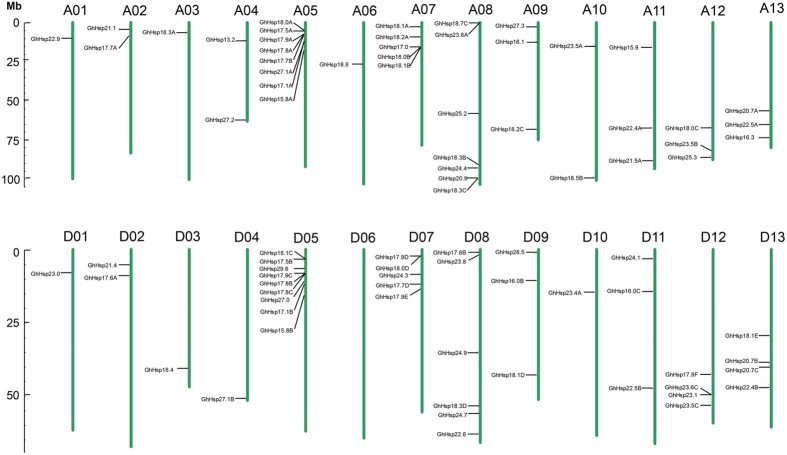
Distribution of the *GhHsp20* family genes on *G. hirsutum* chromosomes. The chromosome number (A1–D13) was shown on the top of each chromosome. The putative *Hsp20* genes are shown on chromosomes 1–13 and from top to bottom. Green bars represent physical maps. Red lines on green bars indicate the locations of *Hsp20* genes in each physical map. The scale is in megabases (Mb).

A total of 82 of the 94 *GhHsp20* genes were physically located on 26 linkage groups (LG) of *G. hirsutum* chromosomes, while 12 genes could not be conclusively mapped to any chromosome, and therefore remain unattributed to any scaffold (Fig. 4). The distribution of *GhHsp20* genes on each chromosome were uneven: chromosomes A01, A02, A03, A04, A06, A09, A10, A11, A12, A13, D01, D02, D03, D04, D09, D10, D11, D12 and D13 contain one to four *GhHsp20* genes, while relatively high densities of *GhHsp20* genes were found in few locations on chromosomes A05, A07, A08, D05, D07and D08. In particular, the *GhHsp20* genes located on chromosomes A05 and D05 were concentrated in the higher end of the arms. Interestingly, closely related genes of the CI subfamily were mainly located on chromosomes A05, A07, D05 and D07, suggesting that expansion of the *GhHsp20* gene family may have occurred via localized or intra-chromosomal duplication.

**Figure 5:**
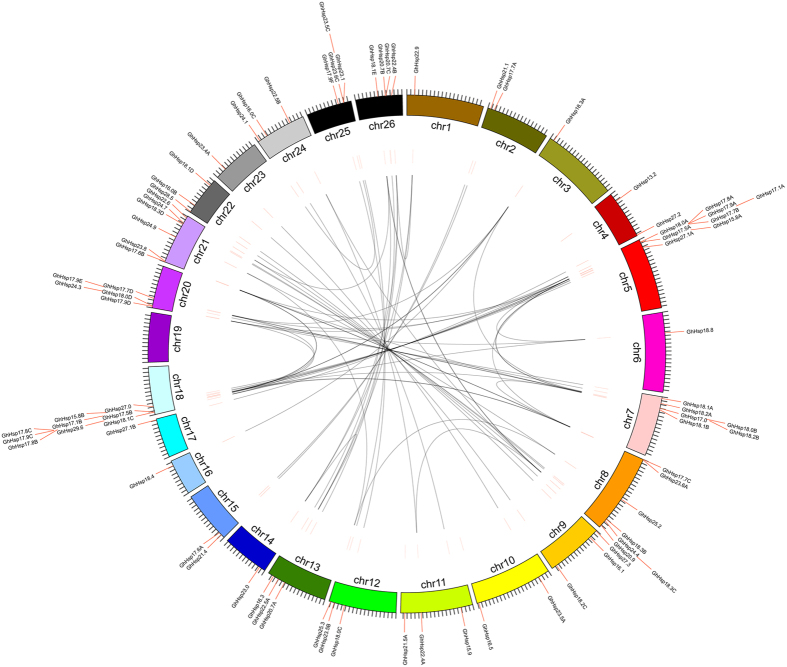
Segmental duplication of *GhHsp20* genes on *G. hirsutum* chromosomes. The approximate distribution of each *GhHsp20* gene is marked with a short red line on the circle. Genome-wide duplicated genes are connected by gray lines.

In general, genome duplication events are thought to have occurred throughout the process of plant genome evolution^18^. Gene duplication events, including tandem and segmental duplications, are thought to play a significant role in the mechanism behind the expansion of the *GhHsp20* gene family^19^. In the current study, the tandem duplication events of 82 *GhHsp20* genes on 26 chromosomes (Supplementary Table 1) were analyzed according to methods described previously^20^, where a chromosomal region of 200 kb containing two or more genes was defined as a tandem duplication event. There were 14 *GhHsp20* genes (*GhHsp17.5A*, *GhHsp18.0A*, *GhHsp17.9A*, *GhHsp17.8A*, *GhHsp18.2B*, *GhHsp18.1B*, *GhHsp18.3C*, *GhHsp20.9*, *GhHsp17.5B*, *GhHsp18.1C*, *GhHsp17.8B*, *GhHsp17.9C*, *GhHsp23.6C* and *GhHsp23.1*) clustered into seven tandem duplication event regions on grape chromosomes A05 (two clusters), D05 (two clusters), A07 (one cluster), A08 (one cluster) and D12 (one cluster) (Supplementary Table 1). Chromosomes A05 (cluster 1 and cluster 2) and D05 (cluster 5 and cluster 6) had two clusters each, indicating a hot spot of *GhHsp20* gene distribution. In addition to the tandem duplication events, segmental duplications were investigated in this study. We searched the genome of *G. hirsutum* for pairs of duplicated regions using MCSCAN (http://chibba.agtec.uga.edu/duplication/mcscan/) to define gene paralogy, and identified 73 paralogous gene pairs (86.2%) among the 94 *Hsp20* genes. Of the mapped *GhHsp20* genes, only seven were located outside of the duplicated blocks, while 90.2% (74 of 82) were located in duplicated regions. Furthermore, 49 genes were involved only two chromosome regions, 13 genes spanned three chromosome regions, 14 genes traversed four chromosome regions, and five genes crossed five chromosome regions (Fig. 5 and Supplementary Dataset 2). Of the 73 *GhHsp20* paralogous gene pairs, 62 remained in conserved positions on segmental duplicated blocks (Fig. 5), providing strong evidence that these 62 paralogous pairs may be derived from segmental duplication events during the evolutionary process, and that gene duplication has made an important contribution to cotton.

The ratio of non-synonymous to synonymous substitutions (*Ka*/*Ks*) is an indicator of the history of selection acting on a gene or gene region^21^. We calculated the *Ka*/*Ks* ratio for each pair of duplicated *GhHsp20* genes to reveal whether Darwinian positive selection was associated with functional divergence after gene duplication. In this study, the *Ka*/*Ks* ratios for 50 of the 73 duplicated *GhHsp20* gene pairs (68.5%) were less than 1 (Supplementary Dataset 2), demonstrating that these genes from *G. hirsutum* experienced relatively rapid evolution following duplication, which lead to functional segregation of *GhHsp20* genes. We further calculated the approximate dates of duplication events with the DnaSP program. The results showed that segmental duplications of *GhHsp20* genes occurred between 4.19 Mya (million years ago) to 535.62. Mya, with an average of 173.16 Mya (Supplementary Dataset 2 and Supplementary Fig. 8), suggesting that the divergence time of this family was before the A- and D-progenitor genomes.

### Expression profiles of *GhHsp20* genes in different tissues

**Figure 6:**
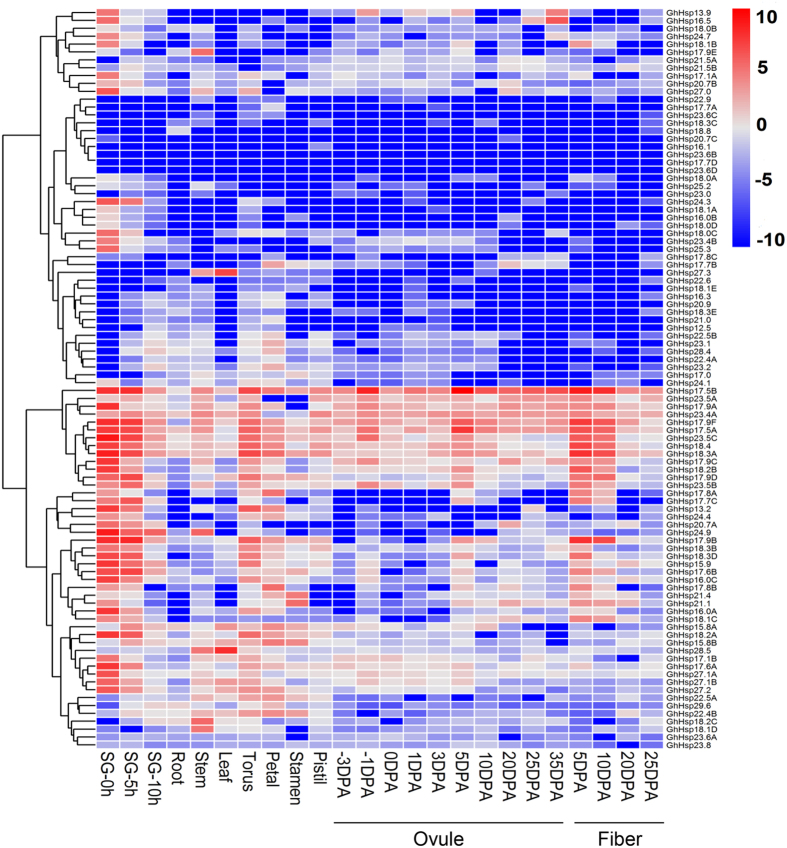
Heat map representation of *GhHsp20* gene expression in different tissues. The tissues used for expression profiling are indicated at the bottom. The genes are shown on the left of the expression bars and the phylogenetic relationship is shown. Scale bars on the bottom right of each heat map represent log_2_-transformed RPKM values. SG 0h, seed germination 0 hour; SG 5 h, 5 hours after seed germination; SG 10 h, 10 hours after seed germination; −3 DPA to 20 DPA indicates −3, −1, 0, 1, 3, 5, 10, 20, 25 and 35 days after pollination.

In order to gain insights into the potential developmental roles of the *GhHsp20* genes, the spatio-temporal expression of individual members of the gene family were investigated using transcriptome datasets of *G. hirsutum* collected from seven different organs: seeds, roots, stems, leaves, torus, petal, stamen, pistil, ovules and fibers. As transcriptome datasets of *G. hirsutum* were available for different tissues, a heat map showing the expression of all the *GhHsp20* genes was generated (Fig. 6). Expression clusters were analyzed by Mev4.6.2 (http://www.tm4.org/mev/), resulting in 14 expression patterns (threshold ≥ 0.5). The results showed ninety percent (82 of 94; 87.2%) of the analyzed *GhHsp20* genes were expressed in all tissues (FPKM  ≥ 1). On the other hand, 12 genes were not expressed in all tested organs and developmental stages, interesting that all the 12 genes belonged to C subfamily, two genes (*GhHsp18.3C* and *GhHsp23.6C*) were from tandem duplication events, seven genes were from segmental duplication events, indicating that these genes may be either functional redundancy in development or pseudogenes. Different *GhHsp20* genes were dominantly expressed in different tissues (Fig. 6). A total of 33 of 94 (35.1%) were highly expressed (FPKM ≥ 50) during seed germination stage. For example, the transcript abundances of mitochondrial localization gene *GhHsp23.5C* were approximately 200 times higher in seed germination than that of the other stages. *GhHsp17.0*, *GhHsp21.4* and *GhHsp21.1* shared high expression levels during stamen development. In total of 10 of 94 (10.6%) *GhHsp20* genes showed primarily expressed in fiber compared with, root, stem, leaf, ovule and flower tissues.

In addition to groups of genes that exhibited similar transcript abundance profiles but were relatively phylogenetically distinct, several phylogenetic clades shared, to a large extent, the same transcript abundance profile. Gene expression patterns can provide important clues for gene function. For example, in tissue expression group 4 (Supplementary Dataset 3), most *GhHsp20* genes were dominantly expressed during seed absorption, indicating that they have a conserved functional role in seed germination. Members of tissue expression group 6 were mainly detectable in leaf tissue, which suggests that they have similar functions in leaf development.

### Expression patterns of *GhHsp20* genes under abiotic stresses and following exogenous hormone treatments

**Figure 7:**
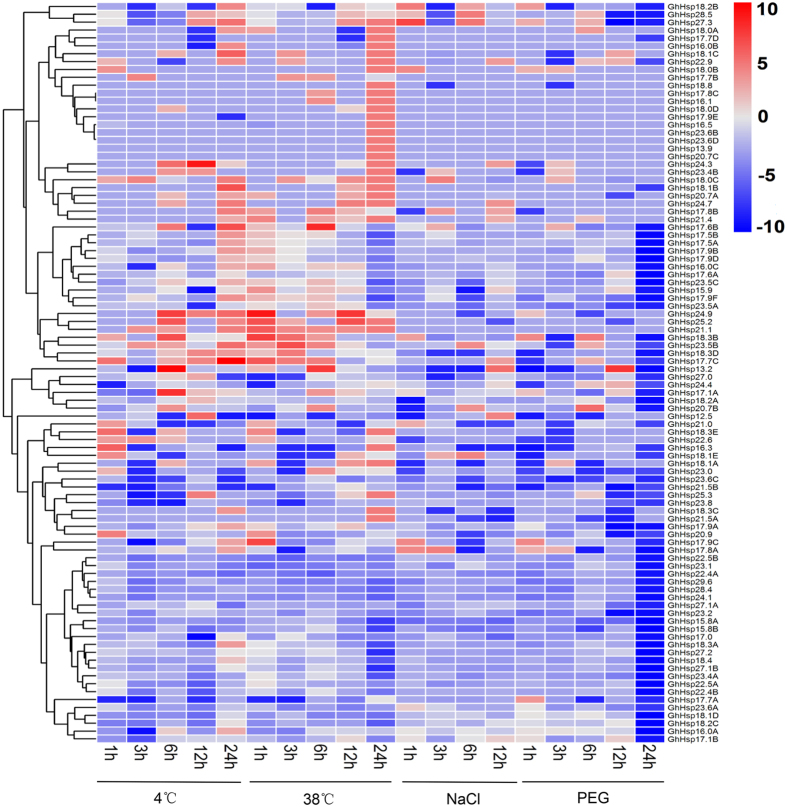
Expression of *GhHsp20* genes under abiotic stress. The genes are shown on the left of the expression bars and the phylogenetic relationship is shown. The abiotic stresses used for expression profiling are indicated at the bottom. Scale bars on the bottom right of each heat map represent log_2_-transformed (Treament_RPKM_/Control_RPKM_) values. 1 h, 3 h, 6 h, 12 h, 24 h indicate hours after treatment.

To explore the responses of *GhHsp20* genes to abiotic stresses, we performed transcriptome sequencing (RNA-seq). The heat map represented of *GhHsp20* genes in response to abiotic stresses such as dehydration, salinity, heat and cold (Fig. 7) All the genes exhibited variations in expression in response to one or more stresses. Of the four treatments, heat stress induced relatively more fluctuations in the transcript abundance of *GhHsp20* than that of the dehydration, salinity and cold. A total of 34 *GhHsp20* genes increased instantly in response to heat treatment, and decreased quickly during the continued heat stress. The highest increase (600-fold) was observed for *GhHsp21.4*. While the expression of *GhHsp17.7B*, *GhHsp20.7B* and *GhHsp23.5A* were slowly increased during the continued heat stress. These results provided an essential clue of several *GhHsp20* genes such as *GhHsp17.9A* and *GhHsp18.2B* as the part of heat stress signaling system, while GhHsp17.7B, *GhHsp20*.7B and GhHsp23.5A proteins played critical roles in protein refolding. Members from group CI (*GhHsp17.8A* and *GhHsp18.3B*) had relatively high expression levels under drought stress, which were also specifically expressed at five dpa, these specifically upregulated genes seem to be more sensitive during fiber development. Over 50% of *GhHsp20* genes were up-regulated under heat stress, whereas, more than a quarter of the *GhHsp20* genes were down-regulated under drought stress conditions. In total 15 *GhHsp20* genes were induced by multiple stresses, while nine *GhHsp20* genes were mainly repressed by all four abiotic stresses. The expression of three *GhHsp20* genes (*GhHsp18.3A*, *GhHsp17.0* and *GhHsp16.0C*) was induced by heat stress but repressed by cold stress. It was noteworthy that some genes were found to be differentially expressed in response to a specific stress treatment at only one time point. For example, *GhHsp24.3* was induced early by heat, while at later time-points only cold was able to induce its expression. Some genes were up-regulated by all stresses at both early and late time points (Fig. 7).

**Figure 8:**
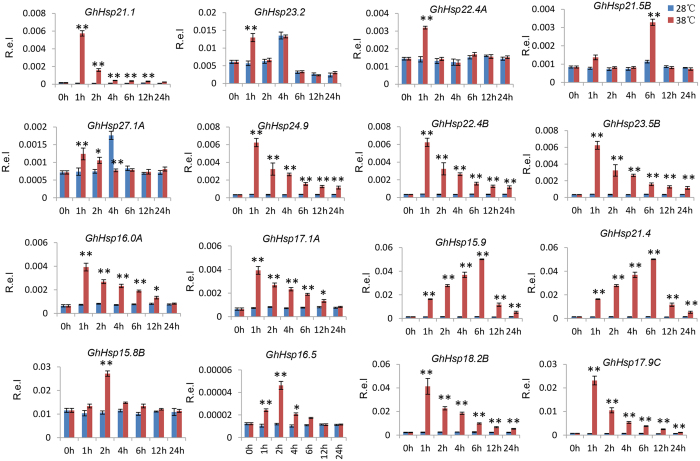
Expression analysis of the selected cotton *GhHsp20* genes in response to 38 °C treatment using qRT-PCR, in comparison to control (28 °C). The mean expression value was calculated from 3 independent replicates. The vertical bars indicate the standard deviation. 0 h, 1 h, 2 h, 4 h, 6 h, 12 h, 24 h indicate hours after treatment. Mean values and standard errors are calculated according the data from three replicates. The asterisk and double asterisks represent significant differences at the levels of 0.05 and 0.01, respectively. R. e. l indicates Relative expression level.

Based on the RNA-seq data (Fig. 7), we also examined the expression of 16 selected *GhHsp20* genes from each branch of the evolutionary tree using qRT-PCR after imposing two stress treatments (heat, and drought) and exposure to three stress-related signaling compounds (ABA, Eth or an oxidative stress inducer [H_2_O_2_]). The transcription levels of 11 *GhHsp20* genes were significantly increased and reached a peak 1 hour after heat treatment (Fig. 8). In particular, the transcript abundance of *GhHsp15.9*, *GhHsp15.5* and *GhHsp21.4* showed an early increase under heat stress. However, the expression of eight genes decreased gradually with time after one hour heat treatment. Notably, *GhHsp27.1A* exhibited an early upregulation but subsequent downregulation compared with control. Drought stress caused upregulation of ten *GhHsp20* genes (Supplementary Fig. 1). Eight genes (*GhHsp21.1, GhHsp24.9, GhHsp23.5B, GhHsp16.0A, GhHsp17.1A, GhHsp15.9, GhHsp16.5* and *GhHsp18.2B*) were upregulated in response to both heat and drought stress. The transcript levels of four *GhHsp20* genes were increased in response to anoxia stress (Supplementary Fig. 2). This further suggested that these common upregulated *GhHsp20* genes possibly participate in cross-talk between signaling pathways to regulate these two stresses.

After ABA treatment, six *GhHsp20* genes were significantly up-regulated. Similarly, eight *GhHsp20* genes were up-regulated by Eth treatment, which caused drastic enhancement of the transcript level (>10 fold) of *GhHsp17.9C*. Four genes were upregulated in response to both ABA and Eth treatments (Supplementary Figs 3 and 4). However, the expression levels of eight genes and nine genes remained unchanged after exposing to ABA and Eth, respectively.

## Discussion

The development of multiple members of a gene family is a long-term natural evolutionary process, and number of the family members reflects the degree of genome amplification and rearrangement during evolution^22^. Studies have shown that gene replication plays an important role in the evolutionary process: *Arabidopsis thaliana* and *Oryza sativa* experienced several genome replications^23^. More and more evidence suggested that Hsp20 proteins play important roles in diverse plant developmental processes as well as various abiotic and biotic stress responses. Benefiting from the availability of genome information, studies have characterized the functions of the *Hsp20* family genes in many plants, including the model plants *Arabidopsis*^24^,^25^ and *Oryza sativa*^26^,^27^. Preliminary analysis of the *Hsp20* gene family has been performed in the *Oryza sativa* and soybean^28^,^29^. Our current work described the identification of *GhHsp20* genes in cotton, including analysis of their structure, evolutionary history, and expression pattern diversity with respect to abiotic stresses.

In this study, we identified 94 *GhHsp20* genes in *G. hirsutum* genome. The number of members in *G. hirsutum* was larger than in *Arabidopsis thaliana* and *Oryza sativa*, suggesting the possibility of a gene gain event during the evolutionary process from diploid to tetraploid. The exon/intron structure is the most ancient level of gene information available^30^. Here we found conservation ofcoding sequences and positions of exon/intron boundaries, although the sizes and sequences of the introns in the coding region were significantly different between the 94 *GhHsp20* genes. The majority of genes in the same phylogenetic subgroup had similar exon–intron structures and motif compositions (Fig. 3). These results suggested that the same or closely related subfamilies have similar motifs and motif distributions, and this supported the previous classification of the *GhHsp20* genes. Genes sharing the same motifs within one subfamily may have the same functions. The above findings may facilitate identification of the functions of *GhHsp20* genes and lead to the discovery of their roles in plant growth and development.

Gene duplication events play a major role in genomic rearrangements and expansions^31^ and are defined as either tandem duplications, with two or more genes located on the same chromosome, or segmental duplications, with duplicated genes present on different chromosomes^32^. In total of 82 of the 94 *GhHsp20* genes were unevenly dispersed on 25 chromosomes of *G. hirsutum*, with the exception of chromosome D06, and seven clusters with two *GhHsp20* genes each were identified (Fig. 4). A number of family members gathered into clusters in certain segments, especially in chromosomes 05, 07 and 08. In our study, we found that a high proportion of *GhHsp20* genes were distributed preferentially in duplicated blocks, suggesting that segmental duplications contributed significantly to the amplification of the cotton *GhHsp20* gene family.

Gene expression patterns are usually closely related to their functions^33^. Analyses of differential expression profiles provide important information with respect to functional specializations of *GhHsp20* genes. Physiologically, seed germination is one of the first developmental processes in plants. In this study, we found that most of the *GhHsp20* genes were activated at the beginning of germination, while *GhHsp18.2C*, *GhHsp17.8C* and *GhHsp18.1D* were expressed at later stages (Fig. 6). The results indicated that most *GhHsp20* genes participated in seed germination, and different *GhHsp20* genes were likely required to allow the plant to specifically expression of Hsp proteins where and when they were required to function in the plant. The mechanisms of action of *GhHsp20* genes in seeding germination required further exploration. Under normal conditions, most *GhHsp20* genes were expressed at low levels in all developmental processes, while only a few genes were highly expressed in specific organs or developmental processes. This finding suggested that *GhHsp20* genes were components of a complex transcriptional network regulating stamen development.

It has been demonstrated that *Hsp20* genes are not only involved in the activation of plant development systems^34^, but also play key roles in the control of plants’ response to environmental stimuli^35^. Since it has been thought that *Hsp20* genes are responsive to plant abiotic stress, we investigated the expression profiles of the *Hsp20* genes in *G. hirsutum* after stress treatments. The data demonstrated that a large number of *Hsp20* genes were rapidly and significantly upregulated within 1 h of heat stress. At least 10 *Hsp20* genes were upregulated by two stress treatments, while nine were down-regulated after all four stress treatments. As shown in Fig. 7, the expression levels of three *GhHsp20* genes (*GhHsp18.3A*, *GhHsp17.0 and GhHsp16.0C*) were increased remarkably by heat stress, but repressed by cold stress. These results indicate that different types of *GhHsp20* genes have different roles in protein refolding under abiotic stresses. Our qPCR results showed that the expression of 16 *GhHsp20* genes was altered in response to at least one of the treatment conditions, suggesting that these *GhHsp20* genes may play important roles in regulating gene expression in response to abiotic stresses. It is remarkable that of the 16 putative *GhHsp20* genes, the expression of *GhHsp17.1A* was induced by heat, drought, H_2_O_2_, ABA and Eth (Fig. 8 and Supplementary Figs 1–4). These expression profiles strongly indicated a divergence in the functions of *GhHsp20* genes in different signal pathways. The functions of these stress-responsive *GhHsp20* genes in abiotic stress resistance will be further characterized in future work.

Until now, only a few *GhHsp20* genes have been functionally characterized in other plant species, and there have been none in cotton. In this study, we have laid a foundation for further identification of the functions of the cotton *GhHsp20* gene family and have provided evidence for the relationship between structure and function in the cotton *GhHsp20* gene family. In addition, our results lay the foundation for understanding the complex mechanisms of abiotic stress signaling controlled by *GhHsp20* proteins in *G. hirsutum*.

## Materials and Methods

### Sequence sources

The sequences of *G. arboreum*, *G. raimondii*, and *G. hirsutum* were downloaded from http://www.phytozome.net/, http://cgp.genomics.org.cn/page/species/index.jsp, and http://mascotton.njau.edu.cn/, respectively. The published Hsp20 protein data for the *Arabidopsis* were obtained from the *Arabidopsis* Information Resource (TAIR release 10, http://www.arabidopsis.org) and the *Oryza sativa* Genome Annotation Project Database (RGAP release 7, http://rice.plantbiology.msu.edu/index.shtml), respectively.

### Identification of cotton Hsp20 sequences

To identify the *GhHsp20* genes in the *G. hirsutum* genome, domain sequences were used as iterative queries to search the *G. hirsutum* genome database using the BlastP program with the Hidden Markov Model (HMM) profile. We used the Hsp20 domain (PF00011, 103 amino acids) as a multiple BLAST query to identify a large number of candidate *GhHsp20* sequences in the *G. hirsutum* database using HMMER software version 3.0^36^. The e-value was set at 1e-10. All Hsp20 candidates were verified using the PFAM program (http://pfam.xfam.org/) to confirm the presence of the Hsp20 domain (PF00011). We identified unique hits and removed redundant sequences from the candidate *GhHsp20* genes according to their corresponding sequences and chromosome locations. In order to improve the precision of the domain analysis, MEME tools (http://meme.nbcr.net/meme/) and the simple modular architecture research tool (SMART)^37^ were used to identify putative domain motifs in the full-length amino acid sequences of cotton *GhHsp20* genes. The molecular weight and isoelectric point of each *GhHsp20* protein were calculated using the online ExPASy program (http://web.expasy.org/compute_pi/).

### Multiple sequence alignments and Intron/Exon structure analysis

The *GhHsp20* sequence was extensively aligned using the ClustalX 2.0 program with the default settings^38^. The *GhHsp20* domains were aligned and the conserved sites were checked manually for their corresponding amino acid residues, which were shaded using DNAMAN software (http://www.lynnon.com/). The alignment was then adjusted manually according to the location of the corresponding amino acids in the Hsp20 motif.

The MEME version 3.5.7 tool was used to identify conserved motifs shared among *GhHsp20* proteins^39^. The following parameter settings were used: maximum number of different motifs to find, 8; optimum motif width, 8 to 100. Subsequently, the MAST program was used to search detected motifs in protein databases^40^.

To obtain the gene structure, the coding regions and genomic sequences of cotton were compared, and the intron distribution pattern and intron splicing phase were derived from the aligned cDNA sequences. A figure showing the *GhHsp20* gene exon lengths was created using the SigmaPlot 10.0 software. The structures were displayed using a gene structure display server^41^.

### Phylogenetic analysis of *GhHsp20* genes

A phylogenetic tree was constructed using the MEGA5.0 software^42^ by the maximum-likelihood (ML) and neighbour-joining (NJ) methods with full predicted protein sequences of cotton, *Arabidopsis* and *Oryza sativa* Hsp20 proteins. For statistical reliability, the nodes of the tree were evaluated by boot- strap analysis with 1000 replicates^43^. Branches with less than 50% bootstrap values were collapsed.

### Mapping *GhHsp20* genes on cotton chromosomes and identification of paralogous genes

A local blast search of the *G. hirsutum* genome sequence was performed to map the physical location of the 94 genes. Mapchart 2.2 software was used to visualize the distribution of the *GhHsp20* genes on the 26 *G. hirsutum* chromosomes.

Comparison of the sequences of paralogous genes based on their evolutionary origins allows a better understanding of the physiological roles of individual genes. Cotton *GhHsp20* gene duplication during evolution was investigated using MEGA software (version 5.0). Evolutionary distances between each *GhHsp20* sequence pair were calculated using ClustalW^44^. For the detection of large segmental duplications, we consulted the duplicated blocks map provided by MCScanXalgorithm (http://chibba.agtec.uga.edu/duplication/mcscan/)^45^. In our analysis, a link was created between two similar genes if (1) alignment between the corresponding proteins gave an E-value lower than 1E-20, (2) the E-value did not exceed 1E-20 times the E-value of the best non-self-hit, in order to restrict the analysis to the closest family members, and (3) at least 50% of the longest sequence was aligned. Finally, a minimum of 6 unduplicated genes were allowed in a block region.

### Calculation of *Ka/Ks* values

Synonymous (*Ks*) and nonsynonymous (*Ka*) substitution rates were calculated according to methods described in a previous study^46^. The *G. hirsutum* Hsp20 duplicated gene pairs were first aligned by the Clustal X2.0 program^47^. *Ks* and *Ka* were then calculated using the DnaSP v5.0 software (DNA polymorphism analysis)^48^. Finally, the *Ka*/*Ks* ratio was analyzed to assess the selection pressure for each gene pair. Generally, *Ka*/*Ks* > 1 signifies positive selection, *Ka*/*Ks* = 1 indicates neutral selection, and *Ka*/*Ks* < 1 shows negative or purifying selection^49^. The date of duplication events was subsequently estimated according to the equation T = Ks/2 r. where “r” is the neutral substitution rate. A neutral substitution rate of 2.6 × 10^−9^ was used in the current study^50^.

### Investigation of the expression pattern of *GhHsp20* genes

Expression data for *GhHsp20* genes was obtained from transcriptome data^50^. These datasets correspond to expression intensities in various tissues and under abiotic stresses. For tissues, gene expression levels were calculated according to FPKM values and the default empirical abundance threshold of FPKM > 1 was used to identify the expressed gene^51^,^52^,^53^. For abiotic stresses, the expression level (FPKM) changes of more than twofold compared with the control was used to identify the up-regulation gene; the expression level (FPKM) changes of little than one half compared with the control was used to identify the down-regulation gene. Expression patterns were clustered by Mev4.6.2 software using the Hierarchical Clustering model (http://www.tm4.org/mev.html).

### Plant materials and treatments

*G. hirsutum* L. acc TM-1 was used to investigate the responses of cotton to abiotic stress treatments. Cotton seedlings were grown in a growth chamber under greenhouse conditions (light/dark cycle: 14 h at 28 °C/10 h at 22 °C; 70% relative humidity). Four-week-old seedlings were treated as follows: To test the response to signaling substances, leaves were sprayed with 100 μM abscisic acid (ABA), 300 mM ethylene (Eth)or 10 mM H_2_O_2_ (ddH_2_O was used as a solvent control). To test the response to drought, the roots of cotton seedlings were irrigated with 20% PEG (ddH_2_O was used as a mock control). To test the response to temperature stress, seedlings were placed in a growth chamber at a high temperature (37 °C) (28 °C was used as a mock control). After being subjected to these stresses, the leaves were collected at the appropriate time points as indicated, frozen in liquid nitrogen and stored at −70 °C.

### RNA extraction and qRT-PCR analysis

The CTAB-acidic phenol extraction method was used to extract the total RNA from cotton^54^. RNA was then treated with DNase I (Invitrogen, http://www.invitrogen.com/) to remove genomic DNA, and 2 μg of total RNA was used for first-strand cDNA synthesis. The primer pairs used for real-time PCR were designed using Beacon Designer 7.0 according to cotton *Hsp20* gene sequences. The annealing temperature was between 56 °C and 60 °C. The cotton histone3 (AF024716) gene was used as the internal control^55^. qRT-PCR was carried out using HiScript Q RT SuperMix (Vazyme, Nanjing, China) with three replicates on an ABI 7500 Real Time PCR System (Applied Biosystems, USA). The amplification parameters were as follows: denaturation at 95 °C for 10 min, 40 cycles of denaturation at 95 °C for 15 s, annealing between 56 °C and 60 °C for 15 s, extension at 72 °C for 15 s. Data were processed using the 2^−ΔΔCT^ method^56^.

## Additional Information

**How to cite this article**: Ma, W. *et al.* Identification and characterization of the *GhHsp20* gene family in *Gossypium hirsutum.* *Sci. Rep.* **6**, 32517; doi: 10.1038/srep32517 (2016).

## Supplementary Material

Supplementary Information

Supplementary Dataset 1

Supplementary Dataset 2

Supplementary Dataset 3

Supplementary Dataset 4
