# Supplementary material for: Identification and characterization of the GhHsp20 gene family in Gossypium hirsutum
Source: Sci Rep. 2016 Sep 1;6:32517. doi: 10.1038/srep32517 (PMC5007520; doi:10.1038/srep32517)
Supplement: Supplementary Information [file srep32517-s1.doc]

Supplementary Information

**Identification and characterization of the** ***GhHsp20* gene family in** ***Gossypium hirsutum***

Wei Ma, Ting Zhao, Jie Li, Bingliang Liu, Lei Fang, Yan Hu ^*^, Tianzhen Zhang ^*^

**Supplementary Fig. 1** Expression analysis of the selected cotton *GhHsp20* genes in response to PEG treatment using qRT-PCR, in comparison to control (H_2_O).

**Supplementary Fig. 2** Expression analysis of the selected cotton *GhHsp20* genes in response to H_2_O_2_ treatment using qRT-PCR, in comparison to control (H_2_O).

**Supplementary Fig. 3** Expression analysis of the selected cotton *GhHsp20* genes in response to ABA treatment using qRT-PCR, in comparison to control (H_2_O).

**Supplementary Fig. 4** Expression analysis of the selected cotton *GhHsp20* genes in response to Eth treatment using qRT-PCR, in comparison to control (H_2_O).

**Supplementary Fig. 5** Phylogenetic analysis of the *Hsp20* gene family from *G. hirsutum*, *Arabidopsis* and *Oryza sativa.*

**Supplementary Fig. 6** Phylogenetic tree of Hsp20 proteins from *G. hirsutum, G. arboreum* and *G. raimondii.*

**Supplementary Fig. 7** Phylogenetic analysis of the *Hsp20* gene family from *G. hirsutum, G. arboreum* and *G. raimondii.*

**Supplementary Fig. 8** Frequency Distributions of duplicated pairs in *G. hirsutum* genome.

**Supplementary Table 1.** Tandem duplication events in the 94 *GhHsp20* genes


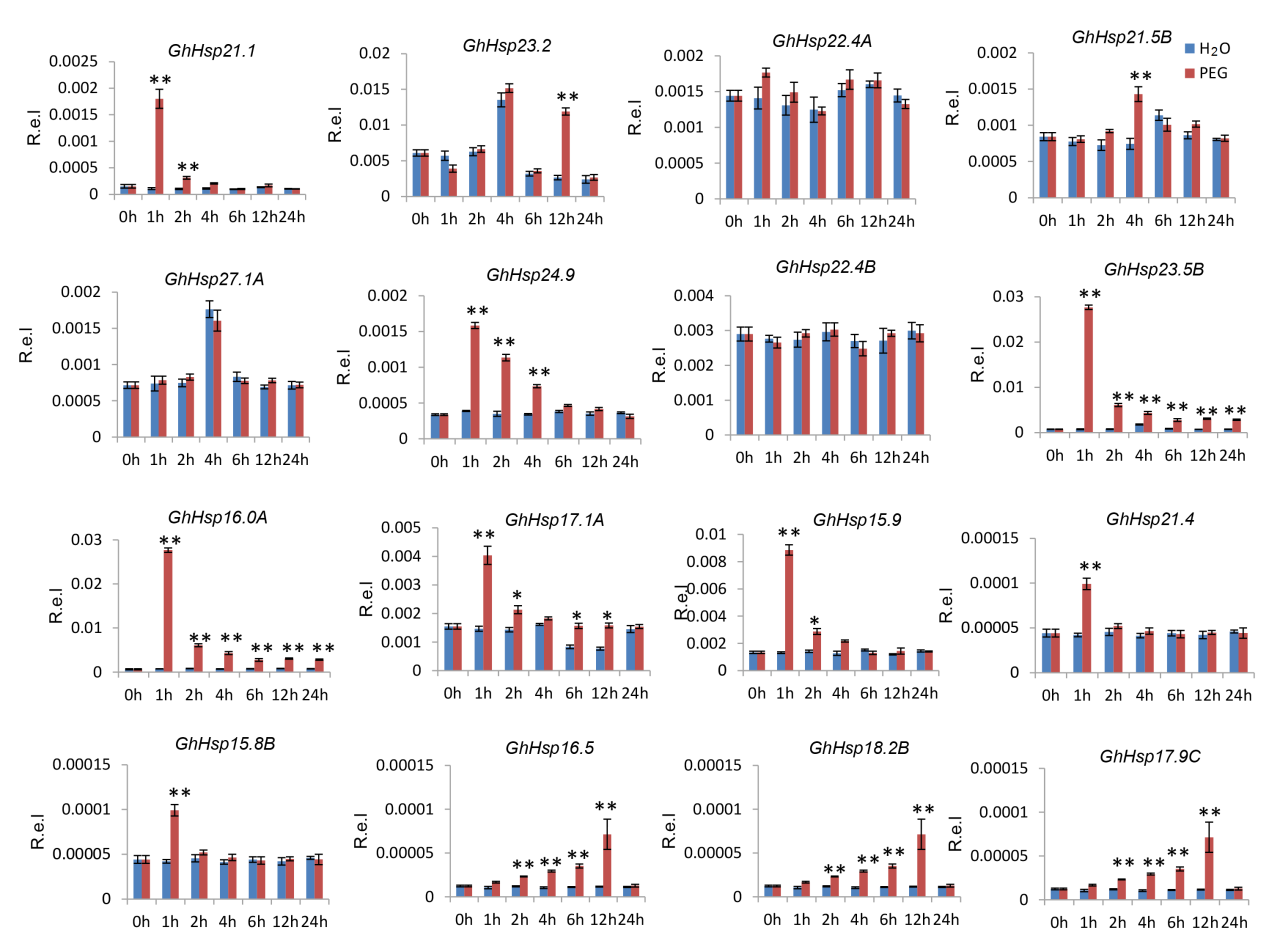


**Supplementary Fig. 1** Expression analysis of the selected cotton *GhHsp20* genes in response to PEG treatment using qRT-PCR, in comparison to control (H_2_O). The mean expression value was calculated from 3 independent replicates. The vertical bars indicate the standard deviation. 0h, 1h, 2h, 4h, 6h, 12h, 24h: hours after treatment. Mean values and standard errors are calculated according the data from three replicates. The asterisk and double asterisks represent significant differences at the levels of 0.05 and 0.01, respectively. R. e. l indicates Relative expression level.


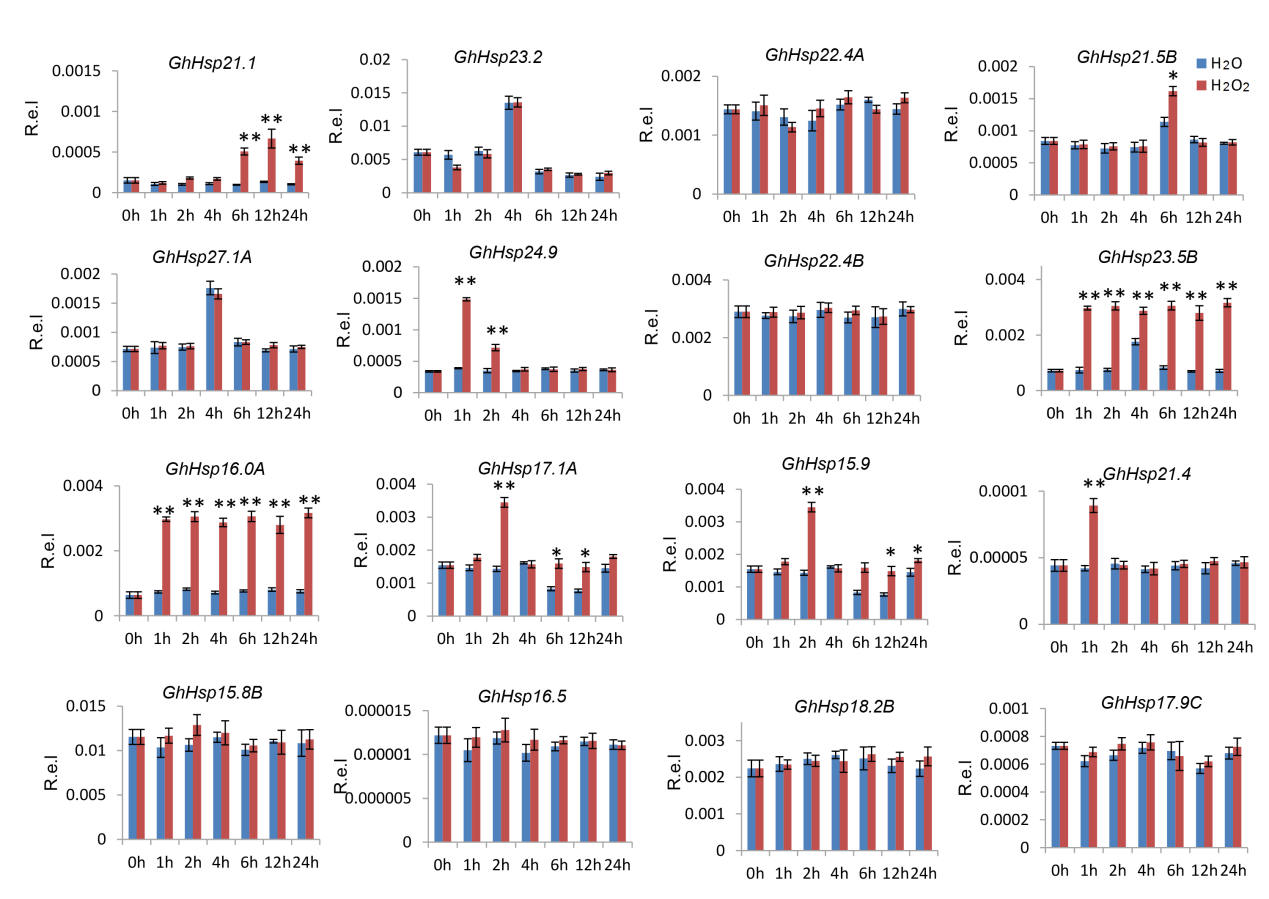


**Supplementary Fig. 2** Expression analysis of the selected cotton *GhHsp20* genes in response to H_2_O_2_ treatment using qRT-PCR, in comparison to control (H_2_O). The mean expression value was calculated from 3 independent replicates. The vertical bars indicate the standard deviation. 0h, 1h, 2h, 4h, 6h, 12h, 24h: hours after treatment. Mean values and standard errors are calculated according the data from three replicates. The asterisk and double asterisks represent significant differences at the levels of 0.05 and 0.01, respectively. R. e. l indicates Relative expression level.


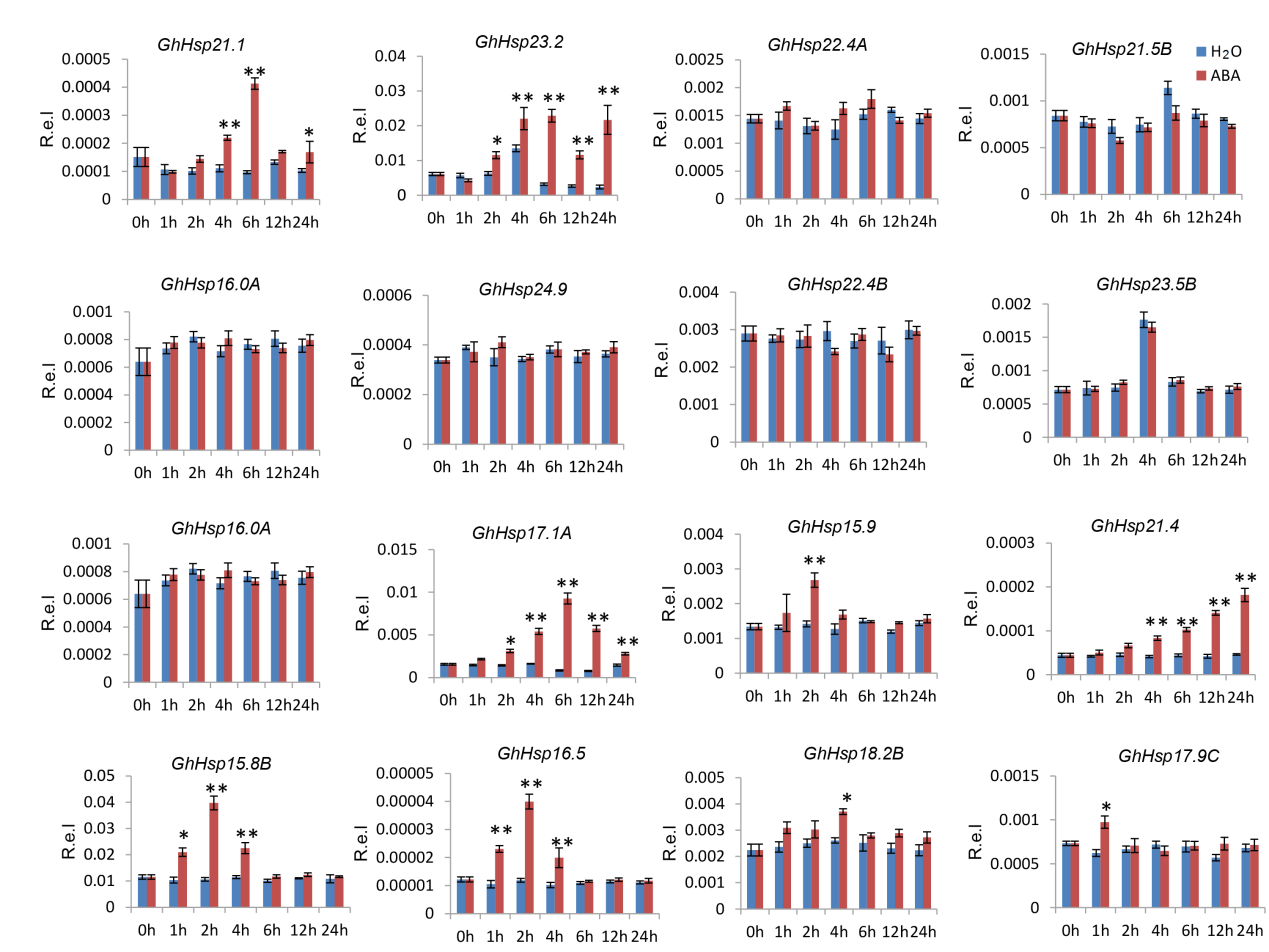


**Supplementary Fig. 3** Expression analysis of the selected cotton *GhHsp20* genes in response to ABA treatment using qRT-PCR, in comparison to control (H_2_O). The mean expression value was calculated from 3 independent replicates. The vertical bars indicate the standard deviation. 0h, 1h, 2h, 4h, 6h, 12h, 24h: hours after treatment. Mean values and standard errors are calculated according the data from three replicates. The asterisk and double asterisks represent significant differences at the levels of 0.05 and 0.01, respectively. R. e. l indicates Relative expression level.


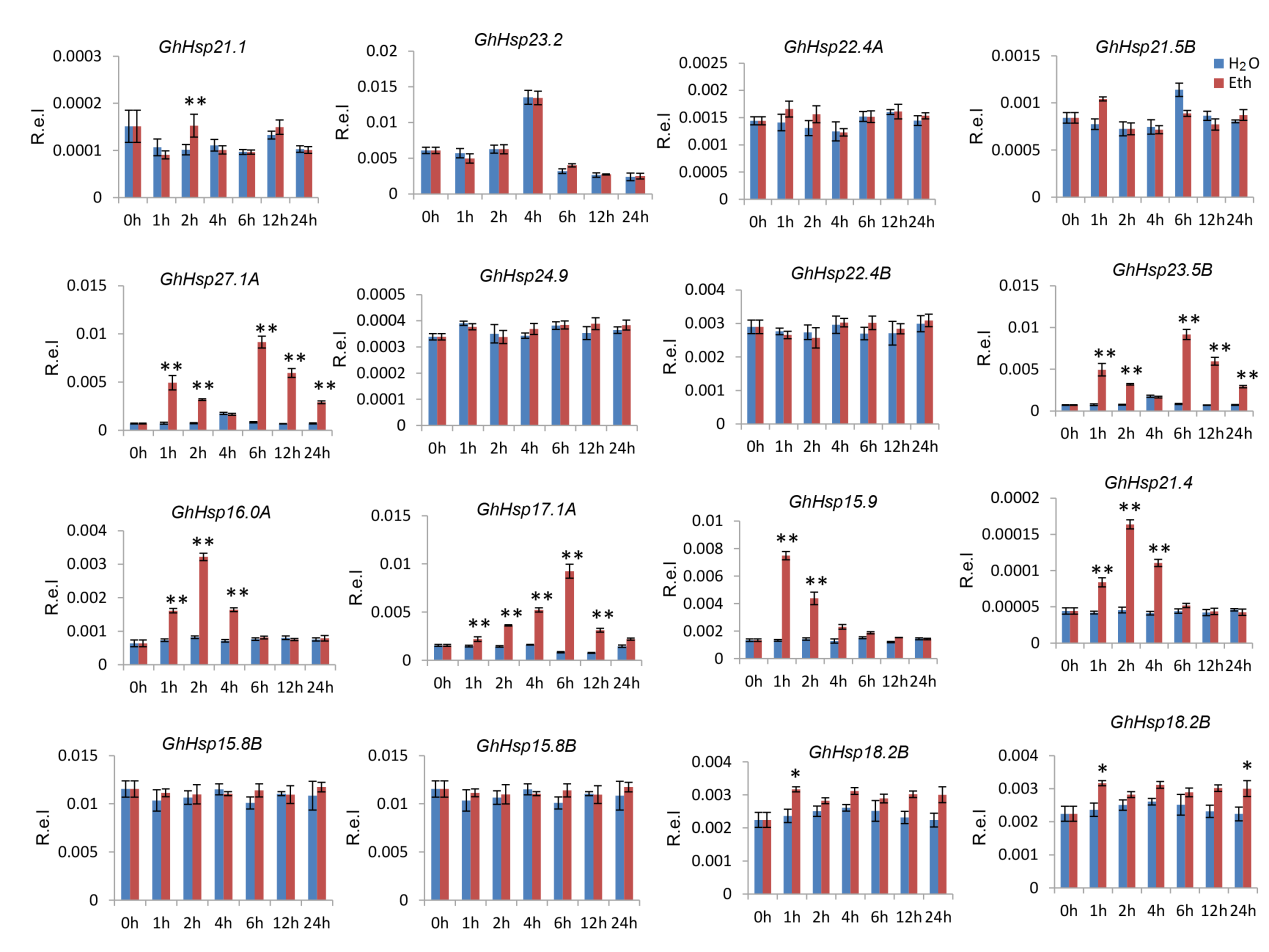


**Supplementary Fig. 4** Expression analysis of the selected cotton *Gh**Hsp20* genes in response to Eth treatment using qRT-PCR, in comparison to control (H_2_O). The mean expression value was calculated from 3 independent replicates. The vertical bars indicate the standard deviation. 0h, 1h, 2h, 4h, 6h, 12h, 24h: hours after treatment. Mean values and standard errors are calculated according the data from three replicates. The asterisk and double asterisks represent significant differences at the levels of 0.05 and 0.01, respectively. R. e. l indicates Relative expression level.

**
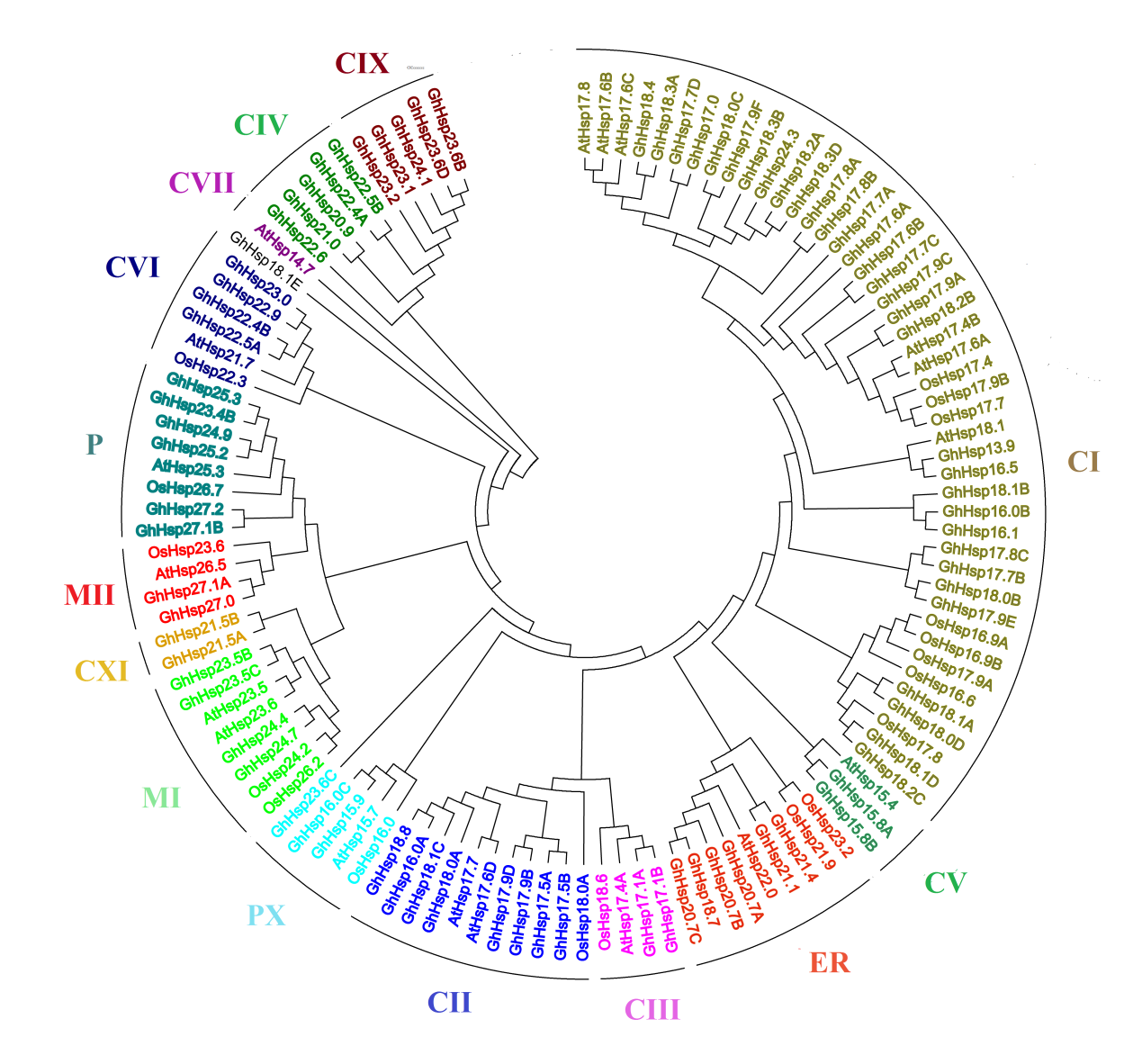
**

**Supplementary Fig. 5** Phylogenetic analysis of the *Hsp20* gene family from *G. hirsutum*, *Arabidopsis* and *Oryza sativa.* The deduced full length amino acid sequences were aligned by ClustalX 2.0 and the phylogenetic tree was constructed using MEGA 5.0 software with the maximum likelihood (ML) method with 1,000 resampling replicates. Each Hsp20 subfamily is indicated by a specific color.

**
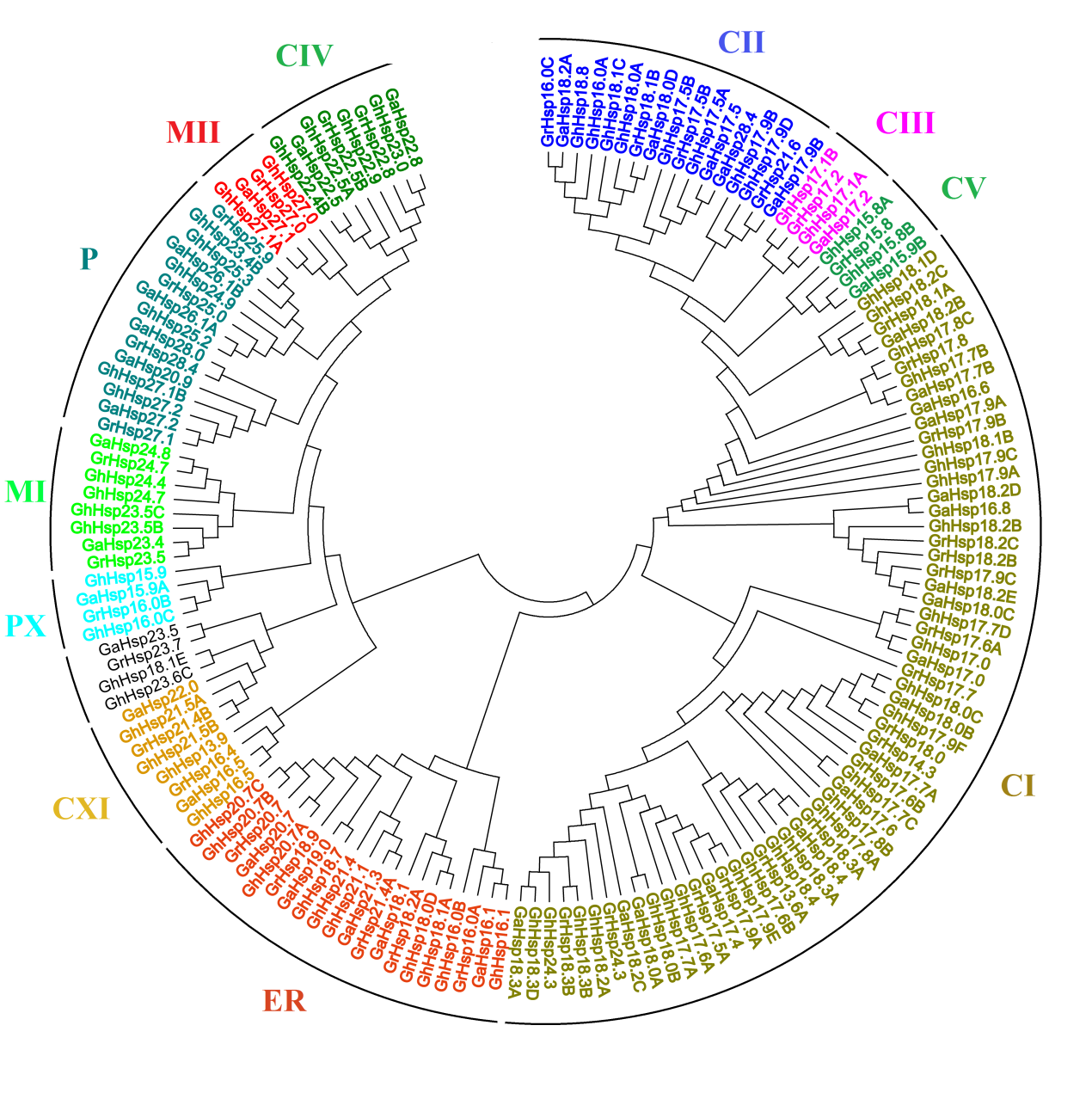
**

**Supplementary Fig. 6** Phylogenetic tree of Hsp20 proteins from *G. hirsutum, G. arboreum* and *G. raimondii.* The deduced full length amino acid sequences were aligned by ClustalX 2.0 and the phylogenetic tree was constructed using MEGA 5.0 by the Neighbour-Joining (NJ) method with 1,000 bootstrap replicates. Each Hsp20 subfamily is indicated by a specific color.

**
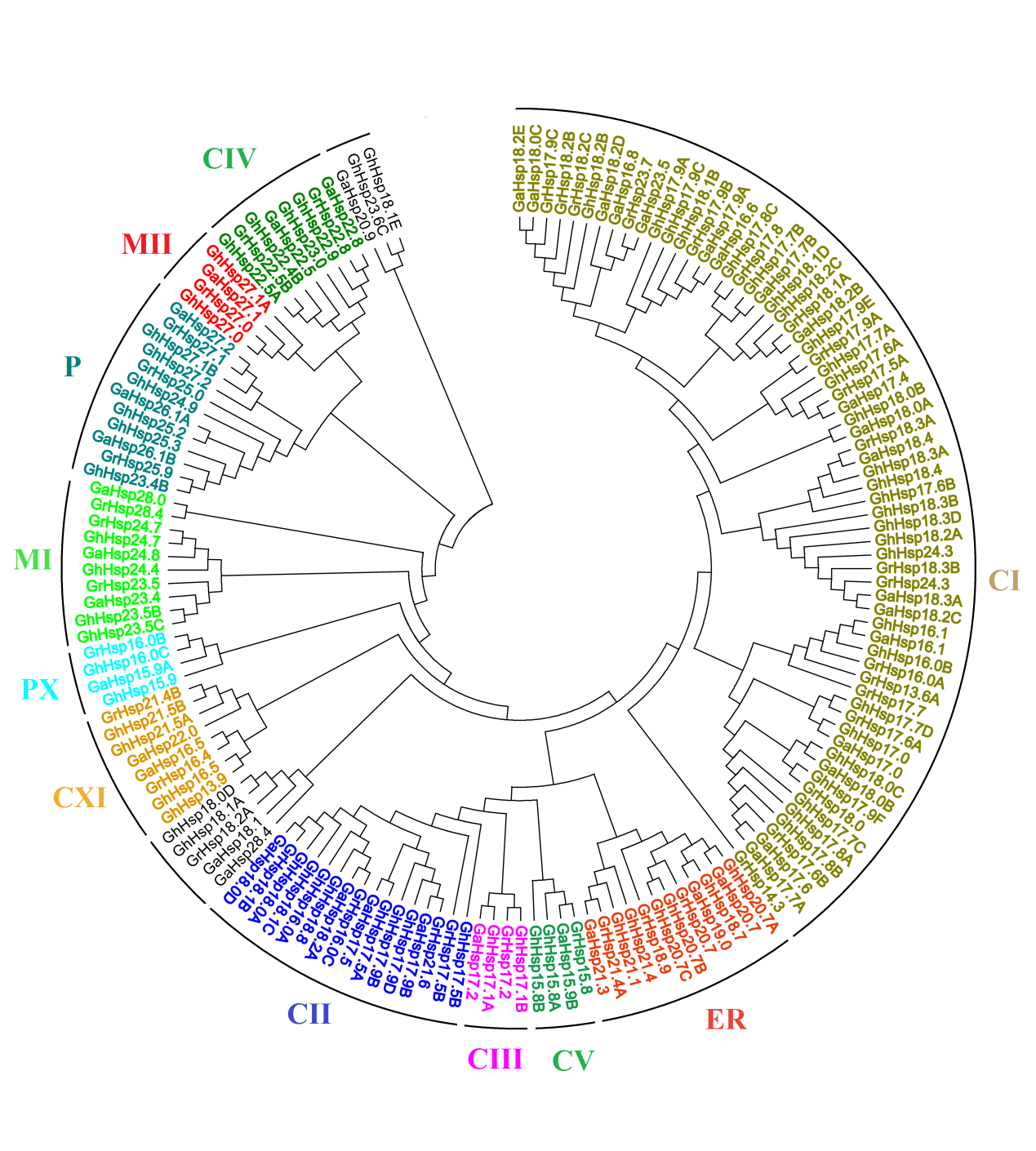
****Supplementary Fig. 7** Phylogenetic analysis of the *Hsp20* gene family from *G. hirsutum, G. arboreum* and *G. raimondii.* The deduced full length amino acid sequences were aligned by ClustalX 2.0 and the phylogenetic tree was constructed using MEGA 5.0 software with the maximum likelihood (ML) method with 1,000 resampling replicates. Each Hsp20 subfamily is indicated by a specific color.

**
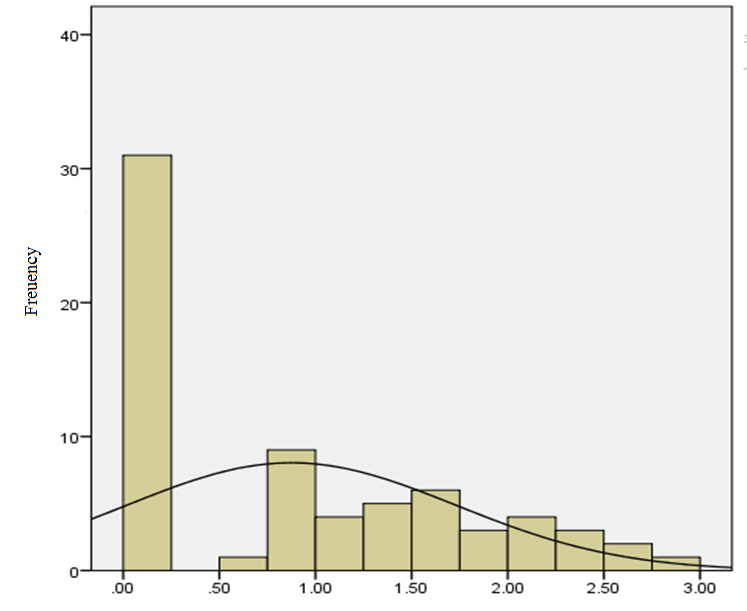
**

**Supplementary Fig. 8** Frequency Distributions of duplicated pairs in *G. hirsutum* genome.

**Supplementary Table 1.** Tandem duplication events in the 94 *GhHsp20* genes

| Cluster number | Gene | Chromosome | Start site (bp) | End site (bp) |
| --- | --- | --- | --- | --- |
| 1 | *GhHsp17.5A* | A05 | 3435796 | 3436266 |
|  | *GhHsp18.0A* | A05 | 3433956 | 3434435 |
| 2 | *GhHsp17.9A* | A05 | 7880231 | 7880701 |
|  | *GhHsp17.8A* | A05 | 7904845 | 7905315 |
| 3 | *GhHsp18.2B* | A07 | 16863712 | 16864191 |
|  | *GhHsp18.1B* | A07 | 16860305 | 16860784 |
| 4 | *GhHsp18.3C* | A08 | 100405244 | 100406186 |
|  | *GhHsp20.9* | A08 | 100402534 | 100403250 |
| 5 | *GhHsp17.5B* | D05 | 3332575 | 3333045 |
|  | *GhHsp18.1C* | D05 | 3330737 | 3331216 |
| 6 | *GhHsp17.8B* | D05 | 7614156 | 7614626 |
|  | *GhHsp17.9C* | D05 | 7603664 | 7604134 |
| 7 | *GhHsp23.6C* | D12 | 49390780 | 49391928 |
|  | *GhHsp23.1* | D12 | 49394637 | 49395342 |
